# *PIK3R1* and *G0S2* are human placenta-specific imprinted genes associated with germline-inherited maternal DNA methylation

**DOI:** 10.1080/15592294.2025.2523191

**Published:** 2025-06-26

**Authors:** Dagne Daskeviciute, Becky Sainty, Louise Chappell-Maor, Caitlin Bone, Sarah Russell, Isabel Iglesias-Platas, Philippe Arnaud, Ana Monteagudo-Sánchez, Maxim V.C Greenberg, Keran Chen, Africa Manero Azua, Guiomar Perez de Nanclares, Jon Lartey, David Monk

**Affiliations:** a Biomedical Research Centre, School of Biological Sciences, University of East Anglia, Norwich, UK; b Neonatology Department, BCNatal - Centre de Medicina Maternofetal i Neonatologia de Barcelona, Institut de Recerca Sant Joan de Déu, Barcelona, Spain; c Neonatal Intensive Care Unit, Norfolk Clinical Research Facility, Norfolk and Norwich University Hospital NHS Foundation Trust, Norwich, UK; d Institut Genetique, Reproduction and Developpement (GReD), CNRS- Universitié Clermont Auvergne-INSERM, Clermont-Ferrand, France; e Universitè Paris Cité, CNRS, Institut Jacques Monod, Paris, France; f Rare Diseases Research Group, Molecular (Epi)Genetics Laboratory, Bioaraba Health Research Institute, Araba University Hospital-Txagorritxu, Vitoria-Gasteiz, Spain; g Department of Obstetrics and Gynaecology, Norfolk and Norwich University Hospital NHS Foundation Trust, Norwich, UK

**Keywords:** Genomic imprinting, placenta, differentially methylated regions

## Abstract

Genomic imprinting is the parent-of-origin specific monoallelic expression of genes that result from complex epigenetic interactions. It is often achieved by monoallelic 5-methylcytosine, resulting in the formation of differentially methylated regions (DMRs). These show a bias towards oocyte-derived methylation and survive reprogramming in the pre-implantation embryo. Imprinting is widespread in the human placenta. We have recently performed whole-genome screens for novel imprinted placenta-specific germline DMRs (gDMRs) by comparing methylomes of gametes, blastocysts and various somatic tissues, including placenta. We observe that, unlike conventional imprinting, for which methylation at gDMRs is observed in all tissues, placenta-specific imprinting is associated with transient gDMRs, present only in the pre-implantation embryo and extra-embryonic lineages. To expand the list of *bona fide* imprinted genes subject to placenta-specific imprinting, we reinvestigated our list of candidate loci and characterized two novel imprinted genes, *PIK3R1* and *G0S2*, both of which display polymorphic imprinting. Interrogation of placenta single-cell RNA-seq datasets, as well as cell-type methylation profiles, revealed complex cell-type specificity. We further interrogated their methylation and expression in placental samples from complicated pregnancies, but failed to identify differences between intrauterine growth restricted or pre-eclamptic samples and controls, suggesting they are not involved in these conditions.

## Key policy highlights

- The promoter of *G0S2* overlaps an oocyte-derived placenta-specific gDMR.
- The promoter of *PIK3R1* isoform 3 is embedded within an oocyte-derived placenta-specific gDMR.
- Allelic methylation at the *PIK3R1* isoform 3 is restricted to trophoblast.
- The orthologues *Pik3r1* and *G0s2* are not imprinted in the mouse placenta.

## Introduction

In mammalian cells, both alleles of autosomal genes typically contribute equally to transcriptional output. However, genes subject to genomic imprinting deviate from this norm, exhibiting monoallelic expression based on the parental origin of the expressed allele. This means some imprinted genes are expressed only from the maternal allele, while others are expressed solely from the paternal allele. Common features of imprinted genes include their dependence on co-organization by imprinting control regions (ICRs) that usually manifest as germline-derived differentially methylated regions (gDMRs) and their association with complex epigenetic regulation. This includes the post-translational histone modifications H3K4me3, observed on the unmethylated allele, while the methylated allele is enriched with heterochromatic marks such as H3K9me3 [1,2]. Currently, around 150 imprinted genes have been identified in humans, frequently showing tissue-specificity, most notably in brain and placenta, some being conserved between species [3–6].

While aberrant imprinted gene expression, via genetic or epigenetic causes, can result in well-characterised imprinting disorders [7], aberrant imprinting has been associated with placenta-mediated pathologies [reviewed in 8], including pre-eclampsia (PE) [9], biparental hydatidiform moles [10], mesenchymal dysplasia [11] and intrauterine growth restriction (IUGR) [12–14]. This is partially because imprinting is widespread in the placenta, with extensive human-specific placenta imprinting having been described by several groups [15–18]. We have recently performed a whole genome scan for imprinted germline DMRs (gDMRs) that are present in the placenta by comparing methylomes of gametes, blastocysts and various somatic tissues [18]. Unlike conventional imprinting, for which methylation at gDMRs is observed in all tissues [15], placenta-specific imprinting is associated with transient gDMRs, present only in the pre-implantation embryo and placenta. This reveals hundreds of potential oocyte-derived gDMRs that survive only in the placenta, many of which orchestrate paternal expression. Moreover, placenta-specific-DMRs can be polymorphic, with some samples being devoid of allelic methylation throughout gestation [10,16,18]. Here, we have reinvestigated the list of partially methylated intervals previously described to be consistent with monoallelic methylation [15] and characterised two novel imprinted genes, the Phopshoinositide-3-kinase regulatory subunit 1 (*PIK3R1*) and G0/G1 switch (*G0S2*), both of which display polymorphic imprinting [19]. Furthermore, we have investigated their potential role in placenta-associated pregnancy complications.

## Material and methods

### Samples

Twelve fetal tissue sets (10–18 weeks gestation; placenta, brain and muscle) were obtained from the Wellcome Trust Human Developmental Biological Resource. A cohort of 92 control placental samples, 32 with corresponding maternal blood/saliva samples, were collected at the Hospital St. Joan de Déu (HSJD; Barcelona, Spain) or Norfolk and Norwich University Hospital (NNUH; Norwich, UK) to assess allelic expression and methylation. Further 35 samples from HSJD were obtained from complicated pregnancies for quantitative expression and methylation studies (see Supplemental Table S1 and S2 for the clinical characteristics of patient samples). For all samples, multiple biopsies from the fetal side around the cord insertion were taken, although for the majority of experiments, only a single site was used. All samples underwent Short Tandem Repeat (STR) analysis (*TH01*, *D13S256* and *D21S1413*) to confirm they were free of obvious maternal contamination.

All mothers provided informed consent for themselves and their child prior to participating in the study. Ethical approval for collecting samples was granted by the Institutional Review Boards at Hospital St. Joan de Déu Ethics Committee (PI35/07) and the University of East Anglia Faculty of Medicine and Health Sciences Research Ethics Committee (ETH2122–0856).

Wild type mouse placentae were produced by crossing *Mus Musculus Molossinus* (JF1) with C57BL/6. Animal husbandry and breeding were conducted according to the institutional guidelines for the care and the use of laboratory animals at Institut Genetique, Reproduction and Developpement (GReD). DNA and RNA extraction and cDNA synthesis were carried out as described previously [15].

### Genotyping and imprinting analysis

#### SNP genotyping

Variants were identified by interrogating the hg19 genome build on the UCSC sequence browser with PCR primers designed to flank SNPs to allow genotype calling by direct sequencing. Sequence traces were interrogated using Sequencher v4.6 (Gene Codes Corporation) or SnapGene 8.0.3 software (GSL Biotech) to distinguish heterozygous and homozygous samples. Heterozygous tissue samples were used for subsequent allelic RT-PCR, methylation-sensitive genotyping and bisulphite PCR (see Supplemental Table S3 for primer sequences).

#### STR genotyping

Highly variable STR markers were amplified on all parental and placenta DNA samples using FAM-labelled primers (see Supplemental Table S3 for primer sequences). To resolve repeat sizes, the PCR amplicons were subject to capillary electrophoresis using POP-7 polymer on an ABI3500 sequencer.

### Analysis of expression

#### Allelic analysis

Expression was analysed in heterozygous samples by RT-PCR and direct sequencing of the resulting amplicons. Imprinting was suggested only if a single base peak was observed at the SNP site in the RT-PCR product of a heterozygous sample. We classified preferential allelic expression as >75% from one allele. Parental origin of expression was determined, when possible, by assessing the maternal genotype. All RT-PCR primers were located in different exons, so that the PCR product crossed a splice site (see Supplemental Table S3 for primer sequences). In addition, RT-PCR was performed on RT-positive and negative samples in order to rule out genomic contamination.

#### Quantitative analyses

Expression levels of *PIK3R1* isoforms were determined by quantitative real-time RT-qPCR with a fluorochrome (SYBR® Green) assay and normalized against *RPL19*. The expression level of *G0S2* was ascertained using TaqMan Real-time PCR assays (Hs00377852_g1) and normalised against *RPL19* (Hs02338565_gH) following manufacture’s recommendations (ThermoFisher, UK). Markers for placental cell-types were determined using datasets from E-MTAB-6701 and subject to SYBR qRT-PCR to identify markers that are specific for pan-trophoblasts (*KRT7*), syncytotrophoblasts (*CGB3*), stromal cells (*COL3A1*), pan-hemopoeitic (*CD45*), Hofbauer (*CD14*) and non-trophoblast fractions (*VIM*)(see Supplemental Table S3 for primer sequences). All assays were run in triplicate in 384-well plates in QuantStudio 5 Real-time PCR System (Applied Biosystems). Dissociation curves were obtained at the end of each reaction to rule out the presence of primer dimers or unexpected DNA species in the reaction. Non-template controls, an interplate control and standard curves from the same serial dilutions of cDNA obtained from pooled placental tissue were included in each assay. Results were scrutinised using the QuantStudio Design and Analysis Software c1.3.1 and Expression Suite Software c1.3 (Applied Biosystems). Amplification plots and automatic baseline and threshold values were individually checked and adjusted where necessary. Only samples with two or more valid readings per triplicate were included. Analysis of the results was performed using the comparative ΔΔCT method. All expression measurements were expressed in a logarithmic scale for normalization, compared to a mix cDNA of all samples, and analysed against clinical values.

### Methylation-sensitive genotyping

Approximately 1 µg of heterozygous genomic DNA was digested with 10 units of HpaII restriction endonuclease for 6 hours at 37°C following our previously published protocols [18,19]. The digested DNA was subject to ethanol precipitation and resuspended in a final volume of 20 µl of water. Approximately 2.5 µl of digested DNA was used in each amplification reaction using BioTaq polymerase (Bioline) for 40 cycles. The resulting amplicons were sequenced and the traces compared to those obtained for the corresponding undigested DNA and maternal samples (see Supplemental Table S3 for primer sequences).

### Bisulphite methylation analyses

#### Allelic PCR

For standard bisulphite conversion, we used the EZ-96 DNA Methylation-Direct (D5023; Zymo Research) following manufacturer’s instructions. Approximately 2.5 μl of bisulphite converted DNA was used in each amplification reaction using Immolase Taq polymerase (Bioline) for 45 cycles and the resulting PCR product sub-cloned into pGEM-T easy vector (Promega) and sequenced with T7 or SP6 primers (see Supplemental Table S3 for primer sequences).

#### Pyrosequencing

**Figure 1.**
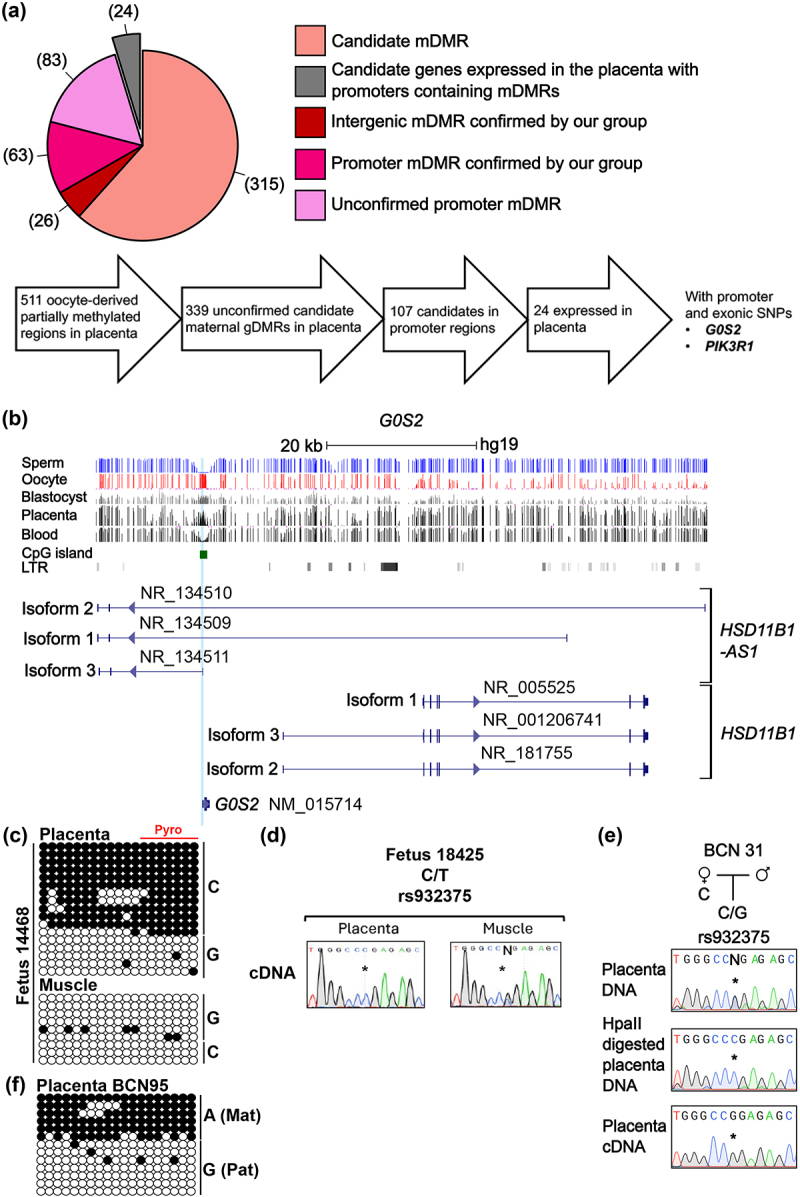
Characterisation of DNA methylation and allelic expression at the *G0S2-HSD11B1-AS1* domain. (a) Venn diagram showing the number of confirmed and uncharacterised candidate placenta-specific maternal DMRs (mDMRs) and a schematic of gene selection. (b) Map of the genomic interval showing complex transcript structure for *HSD11B1-AS1* and the location of *G0S2*. CpG islands are shown in green and the exons of each transcript in blue. DNA methylation profiles observed in sperm, oocyte, blastocysts, placenta and blood methyl-seq datasets are shown. The vertical lines in the methyl-seq tracks represent the mean methylation values for individual CpG dinucleotides. (c) Promoter methylation was confirmed using bisulphite PCR and sub-cloning in Carnegie stage 10 placenta and fetal muscle-derived DNA heterozygous for rs932375. Each circle represents a single CpG on a DNA strand. (•) Methylated cytosine, (o) unmethylated cytosine. Each row corresponds to an individual cloned sequence with associated SNP genotypes. The red line indicates the CpG dinucleotides quantified by pyrosequencing. (d) Sequence traces showing allelic expression profiles in 18-weeks placenta and fetal brain using exonic SNP rs932375. (e) Examples of sequence traces for methylation-sensitive methylation genotyping and allelic RT-PCR products of a 37-week term placenta sample incorporating the rs932375 SNP in *G0S2*. (f) Promoter methylation was confirmed using bisulphite PCR and sub-cloning in a 36-week term placenta sample.

**Figure 2.**
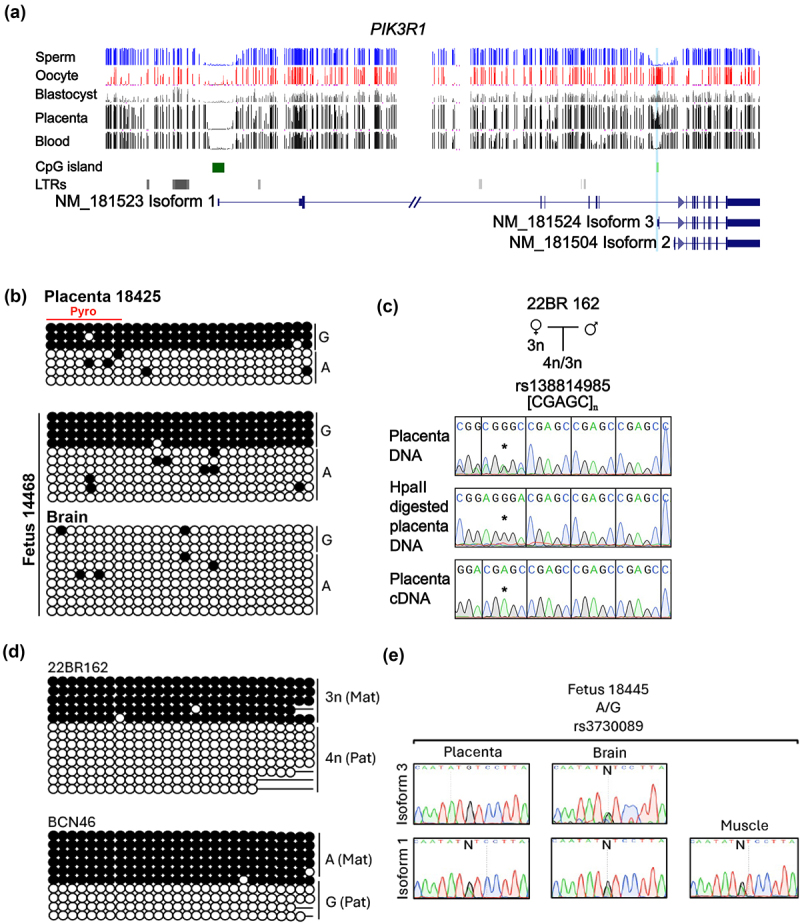
Characterisation of DNA methylation and allelic expression at the *PIK3R1* locus. (a) Map of the genomic interval showing the alternative transcriptional start sites for the *PIK3R1* gene. CpG islands are shown in green and the exons of each transcript in blue. DNA methylation profiles observed in sperm, oocyte, blastocysts, placenta and blood methyl-seq datasets are shown. The vertical lines in the methyl-seq tracks represent the mean methylation values for individual CpG dinucleotides. (b) Promoter methylation was confirmed using bisulphite PCR and sub-cloning in placenta and brain-derived DNA heterozygous for rs2888323 at Carnegie stage 10 (PL14468) and at 18-week gestation (PL18425). Each circle represents a single CpG on a DNA strand. (•) Methylated cytosine, (o) unmethylated cytosine. Each row corresponds to an individual cloned sequence with associated SNP genotypes. The red line indicates the CpG dinucleotides quantified by pyrosequencing. (c) Examples of sequence traces from a term placenta for methylation-sensitive methylation genotyping and allelic RT-PCR products incorporating the rs138814985 in/del for specific for *PIK3R1* isoform 3. (d) Promoter methylation was confirmed using bisulphite PCR and sub-cloning in term placenta samples. (e) Sequence traces showing allelic expression profiles in 18-weeks brain and muscle for *PIK3R1* isoform 1 and 3 using SNP rs3730089.

Standard bisulphite PCR was used to amplify 50 ng of bisulphite-converted DNA, with the exception that one primer was biotinylated. The entire biotinylated PCR product (diluted to 40 µl) was mixed with 38 µl of binding buffer and 2 µl (10 mg/ml) streptavidin-coated polystyrene beads. After incubation at 65°C, DNA was denaturated with 50 µl 0.5 M NaOH. Single stranded DNA was hybridized with 40 pmol sequencing primer dissolved in 11 µl of 90°C annealing buffer, then the mixture was allowed to hybridize at room temperature for 2 minutes. For sequencing, a primer was designed to the opposite strand to the biotinylated primer used in the PCR reaction. The pyrosequencing reaction was carried out on a PyroMark Q24 Advanced instrument. The peak heights were determined using Pyro Q-CpG1.0.9 software (Biotage). In total, 7 CpG dinucleotides were quantified within the *G0S2* gDMR and 8 for *PIK3R1* (locations shown in Figures 1 and 2). Methylation values in plots represent the mean of all CpGs per sample.

### Magnetic-activated cell sorting

Briefly, 5 cm^2^ of a placenta were trimmed to remove decidua and fetal membranes. The samples were minced into ~0.2 cm^3^ pieces and blood vessels removed. Following multiple washed in PBS, the samples were digested in Trypsin solution (25 mL per tube) at 37°C. After 30 minutes digestion was stopped by the addition of 2.5 mL of FBS and the cell suspension passed through a 70 μm cell strainer. Any undigested samples were incubated in Collagenase solution (25 mL per tube) at 37°C. After 30 minutes the cell suspension was passed through a cell strainer and combined with the Trypsin fraction. The cells were washed in PBS and loaded onto a continuous Percoll gradient (5–70%) and centrifuged at 1,600 g (4 accelerate, 0 brake) for 20 minutes at room temperature. The layers between 30% and 55% were removed and washed in PBS before being resuspended in MACs buffer. The samples were split into two fractions, spun and resuspended in 1.5 ml MACs buffer to which 20 μl of anti-human EGFR (BioLegend 352,902) for trophoblasts or anti-human CD90 (BioLegend 328,102) for stromal cells, were added. Washed cell-antibody complexes were incubated with anti-mouse IgG1 secondary microBeads (Miltenyi-Biotec 130–050–601) and loaded onto MS columns on an OctoMACS separator (Miltenyi-Biotec). After three column washes, the positive-bound fractions were collected by removing the MS columns from the magnetics and eluting with MACS buffer using a plunger. This was repeated three times and the cells pelleted for subsequent DNA and RNA isolation. Enrichment was confirmed by marker gene immunostaining and qRT-PCR (Supplemental Figure S1).

### Analysis of public datasets

Methyl-seq datasets were obtained from Gene Expression Omnibus (GEO) or National Biodiversity Data Centre (NBDC) repositories. Datasets for human oocytes (JGAS00000000006), sperm (JGAS00000000006), CD4+ lymphocytes (GSE31263) and preimplantation embryos (JGAS00000000006) were generated by other laboratories, while placenta (GSM1134682) was generated in our lab and published previously [15]. Mouse bisulphite datasets were downloaded from GSE56697, GSE30206, GSE42836. Sequence mapping and methylation calling was performed as previously described [15,18,19]. Placenta-specific cell-type methylation profiles were determined using Illumina Infinium MethylationEPIC arrays datasets visualised on the Placenta Cell Methylome browser (https://robinsonlab.shinyapps.io/Placental_Methylome_Browser/). Placenta single-cell profiles were determined using dataset E-MTAB-6701 [20] with UMAPs visualised using the Human Protein Atlas portal (https://www.proteinatlas.org/humanproteome/single+cell+type).

## Results

### Identification of novel imprinted candidates

We previously screened for differential germline methylation that was maintained in one or more tissues with a profile consistent with an imprinted gDMR. This revealed 511 partially methylated regions inheriting methylation from the oocyte that survived only in the placenta [18]. Of these, 89 had already been confirmed as maternally methylated placenta-specific gDMRs in our previous studies [18–22], with a further 83 verified by others [16,17], leaving 339 unique sequences as unconfirmed candidates (Figure 1a). Here, we revisited these intervals and screened for those regions that map within gene promoters, were expressed in placental cells as revealed by single-cell RNA-seq (scRNA-seq) datasets and contained highly informative SNPs. Phosphoinositide-3-Kinase Regulatory Subunit 1 (*PIK3R1*) and G0/G1 Switch 2 checkpoint (*G0S2*) genes were two genes that fulfilled these criteria and were selected for detailed follow-up characterisation of expression and DNA methylation [19] (Supplemental Figure S2).

### Analysis of allelic DNA methylation and expression at G0S2

The promoter region of *G0S2* (GRCh37/hg19 chr1: 209847680–209849302) was identified as a candidate oocyte-derived gDMR and is located within an intron of the *HSD11B1* antisense transcript (Figure 1b) in publicly available methyl-seq datasets [18]. To confirm that methylation was restricted to one allele, we performed bisulphite PCR on fetal placenta and muscle samples heterozygous for SNP rs932375. The results revealed that the methylation was solely on one allele in placenta (Figure 1c), and absent in muscle-derived DNA. Since the rs932375 SNP is located in the first exon of *G0S2*, it also allows for allelic expression to be determined. Monoallelic expression was observed in placenta, but parental origin could not be assigned due to the lack of a maternal DNA sample. The corresponding fetal muscle sample was biallelically expressed, while no expression was detected in brain (Figure 1d).

To investigate whether the placenta-specific gDMR is maintained in term placenta samples with methylation restricted to the maternal allele, we used bisulphite PCR and/or methylation-sensitive genotyping encompassing rs1815548 or rs932375. Methylation-sensitive genotyping confirms allelic methylation when a heterozygous genomic DNA sample is reduced to homozygosity following digestion with HpaII. Maternal methylation was confirmed in seven term samples, one with bisulphite PCR (Figure 1e,f; Supplemental Table S4) and six by methylation-sensitive genotyping.

To establish if this placenta-specific gDMR orchestrated imprinted expression in term placenta, we interrogated the allelic expression in samples that were heterozygous for rs932375. This revealed two samples with paternal expression, four with maternal expression and two unassigned monoallelic (the parental samples were either unavailable or heterozygous). To ascertain if the placenta samples with unexpected maternal expression possessed low-level maternal contamination, we performed STR genotyping. We did not find any traces of maternal contamination in any sample (Supplemental Figure S3a). Interrogation of scRNA-seq datasets revealed that maternal immune cells expressed *G0S2* much higher than placenta-derived cells. RT-qPCR analysis using a panel of placenta and immune-cell markers revealed that the samples with the highest expression of CD45 lymphocyte common antigen were the same samples that exhibited maternal expression (Supplemental Figure S3b). Therefore, we conclude that despite no trace at the DNA level, which would have required a much higher maternal contamination load for detection, residual maternal-derived immune cells were responsible for the observed maternal expression and that *G0S2* is not randomly monoallelically expressed in different individuals.

### Analysis of allelic DNA methylation and isoform-specific imprinting at PIK3R1

The promoter region of *PIK3R1* isoform 3 (GRCh37/hg19 chr5:67583849–67584928) possesses a DNA methylation profile consistent with being an oocyte-derived gDMR (Figure 2a). To confirm that only one allele is methylated, we performed bisulphite PCR on fetal tissues heterozygous for SNP rs2888323. This revealed monoallelic methylation in a placenta sample with no allelic methylation in the brain (Figure 2b). To confirm that the methylation is solely on the maternal allele, we used bisulphite PCR and/or methylation-sensitive genotyping encompassing rs2888323 and the CGAGC in/del rs138814985 on term placenta samples. Methylation-sensitive genotyping confirmed monoallelic/maternal methylation in 14 samples (Supplemental Table S4) which was corroborated in two samples using bisulphite PCR (Figure 2c,d) .

To characterize the imprinted expression of the various *PIK3R1* transcript isoforms, we assessed the allelic expression in different samples, including first-trimester tissues and term placenta. Isoform-specific RT – PCR primers were designed in unique first exons, spanning alternative splice sites, therefore only allowing the amplification of individual transcripts. Isoform 1 is the most abundant transcript, originating from a unique transcriptional start site ~72 kb upstream of the gDMR. We confirm that isoform 1 is not imprinted in fetal placenta and brain, or 12 term placenta samples (Figure 2a,e) consistent with its promoter being located in an unmethylated CpG island. Using primers that amplify only isoform 3 (note, we could not detect expression for isoform 2), we confirm that this transcript was monoallelically expressed in one fetal placenta sample and biallelic in brain (Figure 2e). Paternal or monoallelic expression was observed in one and eight term placenta biopsies, respectively, with nine being biallelically expressed (Supplemental Figure S4; Supplemental Table S4). This is consistent with both isoform-specific [1,18,23,24] and placenta-specific polymorphic imprinting as reported previously [16–18]. Of the nine samples with biallelic expression for isoform 3, four samples (22BR546, BCN44, BCN54 and BCN217), showed a lack of methylation at the *PIK3R1* DMR (Supplemental Figure S5), while the remaining four possessed a methylation profile consistent with a gDMR (Supplemental Table S4).

### Cell-type specific expression and DNA methylation at the PIK3R1 and G0S2 loci

**Figure 3.**
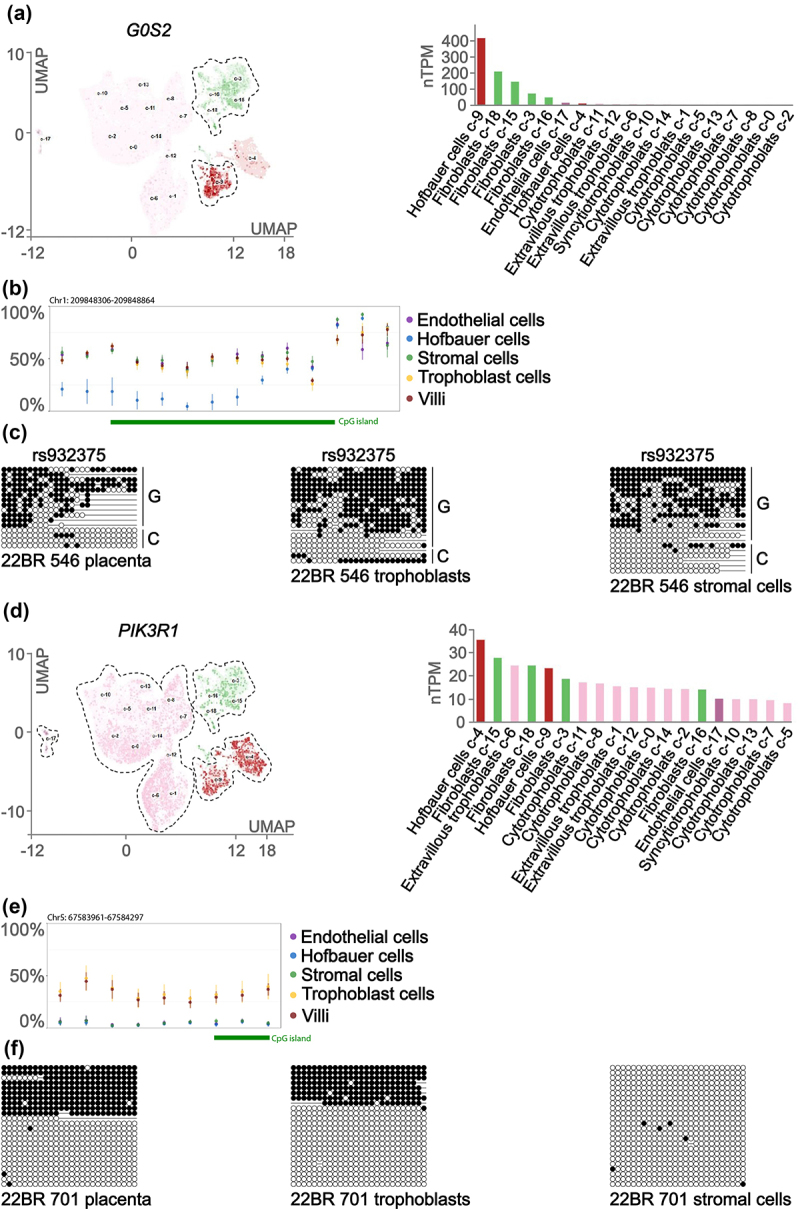
Assessing cell-type specific expression and methylation at the *G0S2* and *PIK3R1* loci. UMAP and bar chart for scRNA-seq showing the cell-type specific expression of (a) *G0S2* and (d) *PIK3R1* in placenta. Illumina methylationEPIC array probes mapping within the (b) *G0S2* and (e) *PIK3R1* gDMR in FAC-sorted placenta cell types. (c,f) Allelic methylation was confirmed using bisulphite PCR and sub-cloning in placenta-derived DNA for both genes in EGFR+ve trophoblasts and CD90+ve stromal cells. Each circle represents a single CpG on a DNA strand. (•) Methylated cytosine, (o) unmethylated cytosine. Each row corresponds to an individual cloned sequence with the parent-of-origin indicated by the genotype of SNP.

Interrogation of published placenta scRNA-seq datasets [20] revealed that *G0S2* is most abundant in the stromal fraction and Hofbauer cells (Figure 3a). Characterization of cell-type specific DNA methylation using the placenta methylome browser showed that the *G0S2* DMR is ~50% methylated in all cell types, except Hofbauer cells, where it is unmethylated. This was confirmed in MAC sorted EGFR+ve trophoblast and CD90+ve stromal cells (Figure 3b,c). Therefore, it is anticipated that imprinted expression should only be observed in stromal cells and not in Hofbauer cells. Unfortunately, the *G0S2* exonic SNP rs932375 was not heterozygous in samples subject to MACs enrichment to confirm this hypothesis. Similar scrutiny of the *PIK3R1* gene revealed ubiquitous expression in all major placenta cell types (Figure 3d). Since these profiles were generated using 10x Genomics scRNA-seq methodologies, sequences are derived from 3’ ends of transcripts and do not allow for isoform-specific expression to be ascertained. Curiously, cell-specific DNA methylation profiling revealed ~50% methylation only in villous trophoblast cells, with endothelial, stromal and Hofbauer cells being unmethylated, suggesting imprinted expression should only be observed in the trophoblast lineages (Figure 3e). These methylation profiles were confirmed in the trophoblast and stromal MAC sorted cells (Figure 3f).

### Methylation profiling reveals polymorphic gDmrs in our cohort

**Figure 4.**
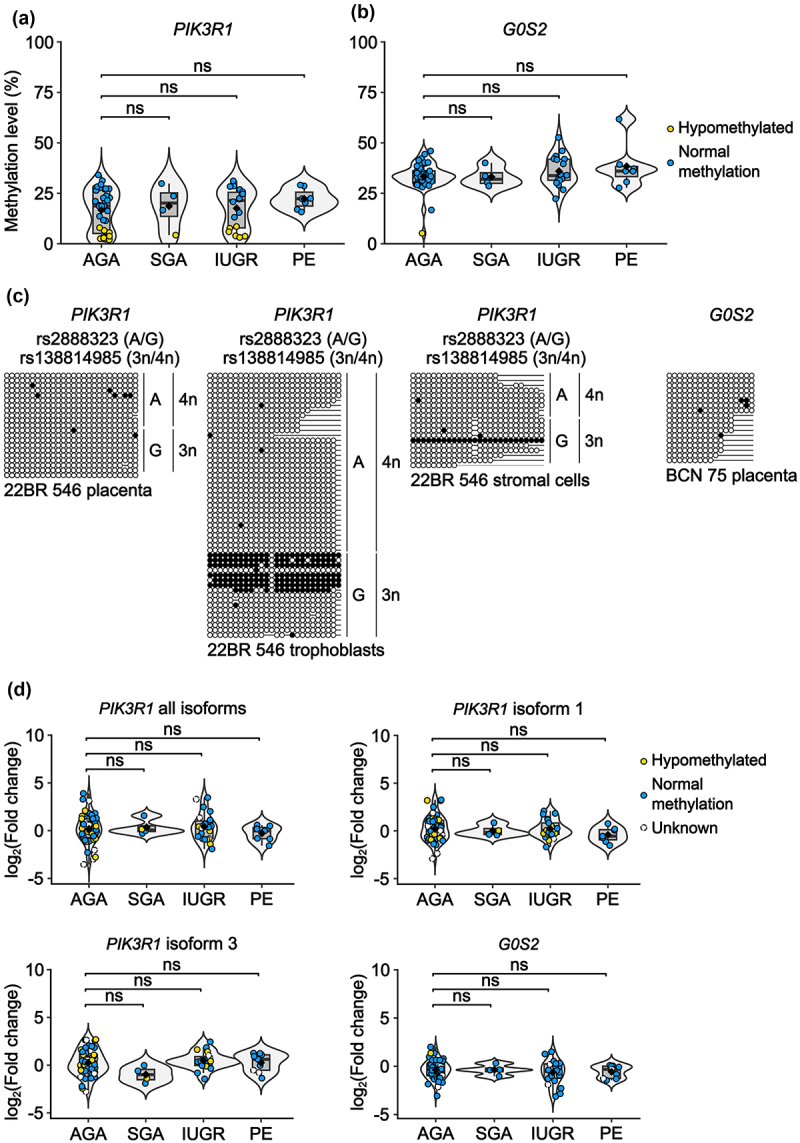
Quantification of *PIK3R1* and *G0S2* methylation and expression in placenta biopsies from complicated pregnancies. The methylation profile for the (a) *PIK3R1* and (b) *G0S2* gDMRs as determined by pyrosequencing of appropriate for gestational (AGA, *n* = 45), small for gestation age (SGA, *n* = 4), intrauterine growth restricted (IUGR, *n* = 19) and pre-eclamptic (PE, *n* = 9) placental biopsies. Results are shown as violin plots containing box plots, which extend from the first to third quartiles (25th to the 75th percentiles), with whiskers indicating 1.5 times the interquartile range below the first or above the third quartiles. Samples outside this range are considered outliers. Each circle represents an individual placental sample. The blue and yellow circles in panels A and B differentiate samples with normal methylation or hypomethylation (<10% methylation) respectively. (c) Severe hypomethylation of representative samples confirmed by bisulphite PCR and sub-cloning. Each circle represents a single CpG on a DNA strand. (•) Methylated cytosine, (o) unmethylated cytosine. Each row corresponds to an individual cloned sequence with the parent-of-origin indicated by the genotype of SNP. (d) RT-qPCR for the expression of *PIK3R1* all isoforms, isoform 1, isoform 3 and *G0S2*. Expression was normalised to the house-keeping gene *RPL19*. All samples were compared to a mixed sample cDNA control allowing for separate comparisons of SGA (*n* = 4), IUGR (*n* = 22) and PE (*n* = 9) groups against AGA controls (*n* = 48). For both methylation and expression analyses, Wilcoxon Mann-Whitney rank-sum test (two-sided) was used to compare mean between groups (ns – not significant).

Polymorphic imprinting has been associated with two different epigenetic scenarios in human placenta samples, either as a consequence of a complete lack of DNA methylation at the defined gDMR interval [16,18,25] or occasionally in the presence of a stable gDMR [14]. To determine if placental samples within our cohort lacked allelic DNA methylation, or if there is variation within samples from complicated pregnancies, we quantified CpG methylation using pyrosequencing (Supplemental Table S5). This revealed that the *PIK3R1* gDMR lacked methylation in 20/72 samples (hypomethylation defined as average methylation <10%), while we observed only one sample lacking DNA methylation at the *G0S2* gDMR (Figure 4a,b). We confirmed the lack of DNA methylation by bisulphite PCR (Figure 4c) which revealed the lack of methylation affected the EGFR+ve trophoblast cells at the *PIK3R1* gDMR. Four placenta samples lacking DNA methylation at *PIK3R1* were heterozygous for rs3730089 and associated with biallelic expression (Supplemental Figure S5). Importantly, the cases lacking allelic methylation were observed in both control placental samples with birthweights appropriate for gestation age (AGA) and complicated pregnancies (IUGR, SGA and PE), indicating that lack of methylation is not associated with abnormal outcomes. Furthermore, we failed to detect differences in mean methylation between placental samples from control and complicated pregnancies.

### Expression profiling in placenta biopsies from complicated pregnancies

We have previously shown that significant differences in expression can be independent of imprinted DMR status [14,26,27]. To determine if absolute expression levels of *G0S2* and *PIK3R1* isoforms 1 and 3 are associated with complicated pregnancies, we performed quantitative RT-PCR. To identify significant differential expression between control AGA samples and IUGR, SGA or PE, we carried out an unpaired Wilcoxon Mann–Whitney rank-sum test (two-sided) (*p* < 0.05). This revealed that there was no significant difference between groups (Figure 4d).

### Placenta-specific Pik3r1 and G0s2 gDmrs are absent in mice

**Figure 5.**
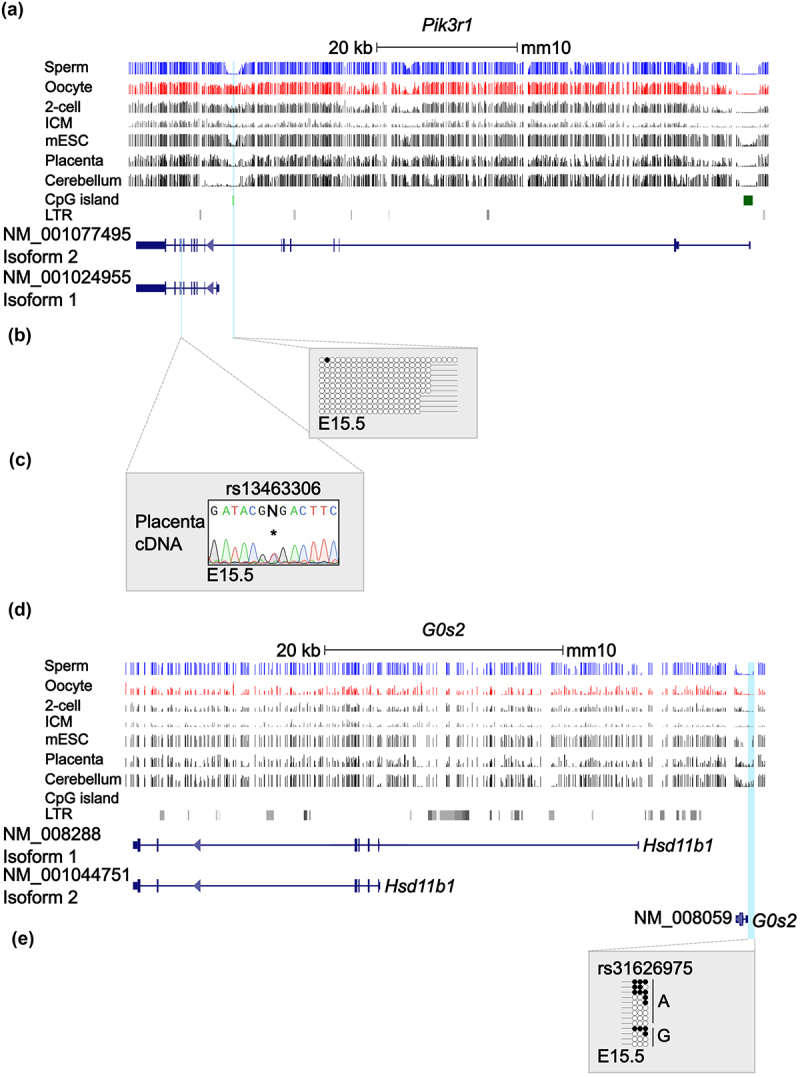
Analysis of allelic methylation and expression for *G0s2* and *Pik3r1* in mouse placenta. (a, d) Maps of the genomic intervals showing the structure of the *Pik3r1* and *G0s2* loci in the mouse genome. CpG islands are shown in green and the exons of each transcript in blue. DNA methylation profiles observed in sperm, oocyte, 2-cell blastomere, inner cell mass (ICM) of blastocysts, mouse embryonic stem cells, placenta and cerebellum methyl-seq datasets are shown. The vertical lines in the methyl-seq tracks represent the mean methylation values for individual CpG dinucleotides. (b, e) Bisulphite PCR and sub-cloning of the orthologous human gDMRs in the placenta of intersubspecific C57BL6 ×JF1 mouse cross. Each circle represents a single CpG on a DNA strand. (•) Methylated cytosine, (o) unmethylated cytosine. Each row corresponds to an individual cloned sequence with the parent-of-origin indicated by the genotype of SNP. (c) Electropherogram of an RT-PCR product showing biallelic expression of *Pik3r1* from mid-gestation mouse placenta.

The majority of the human placenta-specific imprinted gene orthologues are devoid of methylation in the mouse placenta [15,18]. Utilising both mouse methyl-seq data and direct confirmation in placenta-derived DNA from inter-species crosses, we do not detect any maternal methylation for the *Pik3r1* (Figure 5a,b) and *G0s2* (Figure 5d,e) at the interval orthologues to the gDMRs. RT-PCR revealed biallelic expression of *Pik3r1* (Figure 5c) and absence of *G0s2* expression in B6 × JF1 mouse placenta, reinforcing that this phenomenon is not observed in mice.

## Discussion

Genomic imprinting was first reported in the mid-1980’s, and since then, the identification of imprinted genes has been an intensive area of research. This is partly due to the fact that the protein products encoded by imprinted genes have been shown to be important for development and involved in a range of human diseases [8]. To date, there are approximately 150 imprinted genes in humans, but this number is likely much higher as tissue-specific monoallelic regulation is underestimated [28]. The placenta is particularly unique as hundreds of maternally methylated gDMRs have been identified, but not all have been shown to dictate allelic expression [15–18]. This is further complicated by the fact that, unlike in somatic tissues, imprinting can be polymorphic between individuals [14,16,18], whether this is due to polymorphic gDMR establishment or maintenance remains to be determined. To assist in cataloguing imprinting in the human placenta, we continued to characterise allelic DNA methylation and expression in the placenta to identify two novel imprinted genes, *G0S2* and *PIK3R1*.

We confirm that, in the absence of maternal contamination, *G0S2* expression originates from the paternal allele, with the maternal allele silenced by DNA methylation. Previous studies by Hamada and colleagues could not ascertain the imprinting status of *G0S2* in RNA-seq datasets because of maternal contamination. In our sample set, biopsies with no detectable expression of maternal immune markers were paternally expressed, whereas those with residual immune cell contamination (not detectable by STR analysis) were erroneously classified as maternally expressed. This highlights that great care in sample preparation and data interpretation is required when analysing allelic expression in placental samples. Previous cases of maternal expression have been associated with maternal decidual contamination in mouse placentae (e.g., *Dcn* and *Gatm*) [29,30]. While some maternally expressed genes, highlighted by *Tfpi2* [3,31], show maternal expression in trophoblasts and in maternal decidua, many simply result from maternal contamination. Proudhon and Bourc’his outlined a genetic strategy to distinguish true maternal expression from maternal contamination based on the dam’s genotype using inbred strains of mice [32]. If heterozygous mothers are crossed with homozygous fathers, maternal contamination will always manifest as biallelic expression. Unfortunately, all mothers in our study were homozygous for the *G0S2* SNPs used.

The structure of *G0S2* locus resembles several imprinted domains with characteristic allele-specific polyadenylation immediately upstream of a gDMR resulting in imprinting [33,34]. We could not determine if the shorter (NR_134511.1) or full-length (NR-124509.1) *HSD11B1-AS1* transcripts are also imprinted as there are no polymorphisms that would allow for allelic discrimination. The *G0S2* gene encodes a 103 amino acid adipose triglyceride lipase inhibitor which has links to cell proliferation [35]. Although studies of this gene in placenta are scarce, Barrett and colleagues assessed if *G0S2* was involved in gestational diabetes mellitus (GDM), as resulting babies are born large for gestational age with elevated body fat, which may, in part, be due to placenta lipases transferring lipids from mother to fetus [36]. In our data, no difference in expression was observed when comparing samples from complicated and uncomplicated pregnancies. Therefore, despite being a promising candidate, it seems unlikely that *G0S2* plays a major role in regulating placenta-mediated IUGR or GDM.

In addition to *G0S2*, we also describe the isoform-specific imprinting of *PIK3R1* for transcripts originating in the vicinity of a maternally methylated gDMR, whilst a longer isoform from an upstream promoter is biallelically expressed. This gene structure has also been reported for many imprinted genes and is likely related to the co-transcriptional dependency for establishing oocyte-derived intronic CpG island methylation [37]. *PIK3R1* encodes the p85α regulatory subunit of the Class 1A phosphoinositide 3-Kinase (PI3K). PI3K is an obligate heterodimer, with an SH2 domain-containing a regulatory subunit (p85α) and a catalytic subunit (p110) [38]. The p85α subunit mediates binding activation and localisation of the catalytic p110 subunit, with p85α central to the metabolic actions of insulin and insulin-like growth factors that ultimately regulate cellular growth, migration, metabolism and protein synthesis [39,40]. Interestingly, *PIK3R1* adds to the list of imprinted genes in the IGF signalling pathway, which includes *IGF2* [41, *]IGF2R* [1] and *GRB10* [42], with *IGF1R* harbouring a maternal gDMR, although imprinted expression has not been reported [15]. Consistent with a role in mediating growth, *PIK3R1* mutations in humans result in SHORT syndrome [43,44], a developmental disorder characterised by severe defects in fetal growth, although no parent-of-origin effects have been reported in the presentation of this disease. SHORT syndrome has significant phenotypic overlap with Silver-Russell syndrome, an imprinting disorder associated with diminished *IGF2* expression [45], which is considered as a differential diagnosis for SHORT syndrome [46]. Studies in mice confirm a role for *Pik3r1* in regulating fetal development, with *Pik3r1*^WT/Y657^ heterozygotes being viable but smaller at embryonic day E15.5 compared to wild-type litter mates, while homozygous *Pik3r1*^Y657/Y657^ are embryonic lethal at E11.5 [47]. Importantly, placental studies have demonstrated that although no significant difference in total placental mass was observed, a significant reduction of the vascularisation of the placental exchange region was seen in *Pik3r1*^*WT/Y657*^ heterozygous mice at E15.5 [48]. In addition, another member of the Class IA PI3Ks, p110α, also regulates fetal and trophoblast development by regulating nutrient supply and growth [49]. In line with this, reduced expression of *PIK3R1* has been reported in preterm placentas [50], although individual isoforms were not quantified separately. We did not observe differential expression of individual *PIK3R1* isoforms in placenta samples from complicated pregnancies in our cohort that varied in gestational age. These findings suggest that *PIK3R1* could be pivotal in the formation and function of the early placental tissues, highlighting the need to follow-up our observations at earlier developmental time points to ascertain the role of *PIK3R1* in developmental angiogenesis and early onset IUGR.

In summary, we identify two new imprinted genes in the human placenta, which like many imprinted genes in this extra-embryonic tissue, are not imprinted in mice. Both genes are subject to cell-type specific expression and epigenetic regulation, as well as polymorphic imprinting, but additional studies are needed to decipher their roles in placenta-mediated pathologies.

## Supplementary Material

Supplemental Material

## Data Availability

The molecular data that support the findings of this study are available from the corresponding author [DM], upon reasonable request. However, patients did not consent to share meta-data.
