# Supplementary material for: PIK3R1 and G0S2 are human placenta-specific imprinted genes associated with germline-inherited maternal DNA methylation
Source: Epigenetics. 2025 Jun 26;20(1):2523191. doi: 10.1080/15592294.2025.2523191 (PMC12203861; doi:10.1080/15592294.2025.2523191)
Supplement: Supplemental Material [file KEPI_A_2523191_SM1272.zip › Supplementary files/Supplemental Online Material caption.docx]

**Supplemental Online Material**

**Supplemental Figure 1. qRT-PCR enrichment of MACS fractions.** RT-qPCR, normalised to the *RPL19* house-keeping gene, for the trophoblast marker *KRT7* and the stromal marker *VIM*, in positive and negatively enriched fractions.

**Supplemental Figure 2.** Molecular workflow for placenta samples. DNA samples were subject to SNP genotyping and methylation profiling, while expression was assessed by RT-PCR.

**Supplemental Figure 3. Assessment for maternal contamination in placenta samples.** (A) Examples of capillary electrophoresis for STR markers showing maternal and placenta genotypes. The dashed areas indicate the position of the non-inherited maternal allele in each sample. (B) RT-qPCR for placenta-cell type-specific markers in samples with maternal and paternal *G0S2* expression. RT-qPCR marker assessed include: pan-trophoblasts (*KRT7*), syncytotrophoblasts (*CGB3*), stromal cells (*COL3A1*), pan-haemopoietic (*CD45*), Hofbauer (*CD14*) and non-trophoblast fractions (*VIM*). Samples BCN31 and 23BR128 are paternally expressed while 21BR311, 21BR430 and 21BR432 are maternally expressed.

**Supplemental Figure 4. Allelic expression of *PIK3R1* isoforms in term placenta.** Sequence traces determining the allelic expression of *PIK3R1* isoforms 1 and 3 using SNP rs3739989.

**Supplemental Figure 5. Allelic bisulphite PCR and expression profiling in term placenta samples lacking *PIK3R1* methylation.** Promoter methylation was assessed using bisulphite PCR and sub-cloning in placenta samples with hypomethylation revealed by pyrosequencing. Each circle represents a single CpG on a DNA strand. (•) Methylated cytosine, (o) unmethylated cytosine. Each row corresponds to an individual cloned sequence with associated SNP genotypes. (B) Sequence traces showing biallelic expression of *PIK3R1* isoform 3 using SNP rs3730089.

**Supplemental Table 1.**

Individual clinical characteristics of the placenta samples used for qRT-PCR and pyrosequencing.

**Supplemental Table 2.**

Summary statistics for the clinical characteristics of the placenta samples used for qRT-PCR and pyrosequencing.

**Supplemental Table 3.**

List of PCR primers used in this study.

**Supplemental Table 4.**

The number of heterozygous samples used to determine allelic methylation and expression for *G0S2* and *PIK3R1* in fetal and term datasets.

**Supplemental Table 5.**

Pyrosequencing results for individual CpGs within the *G0S2* and *PIK3R1* gDMRs.
