# Supplementary material for: PIK3R1 and G0S2 are human placenta-specific imprinted genes associated with germline-inherited maternal DNA methylation
Source: Epigenetics. 2025 Jun 26;20(1):2523191. doi: 10.1080/15592294.2025.2523191 (PMC12203861; doi:10.1080/15592294.2025.2523191)
Supplement: Supplemental Material [file KEPI_A_2523191_SM1272.zip › Supplementary files/Supplemental_Tables_1_2.docx]

**Supplemental Table 1**. Individual clinical characteristics of the placenta samples used for qRT-PCR and pyrosequencing. Term (37 > weeks), PTL – preterm late (35 to 37 weeks), PTM – preterm moderate (32 to 35 weeks), PTE – preterm extreme (<32 weeks).

| **PL_ID** | **Gestational age** | **Child's phenotype** | **Sex** | **Birth weight (g)** | **Mother’s age** | **Mother's hight (mm)** | **Mother's weight (kg)** | **Mother's gained weight (kg)** | **Pyrosequencing** | **qRT-PCR** |
| --- | --- | --- | --- | --- | --- | --- | --- | --- | --- | --- |
| 1 | Term | AGA | Female | 3100 | 33 | 156 | 60 | 23 | Yes | Yes |
| 2 | Term | AGA | Female | 3180 | 32 | 165 | 79 | 8 | Yes | No |
| 3 | Term | AGA | Male | 2555 | 25 | 165 | 88 | 9 | No | Yes |
| 6 | Term | AGA | Female | 2200 | 19 | NA | NA | NA | Yes | Yes |
| 7 | Term | AGA | Female | 2240 | 26 | 154 | 48 | 14 | Yes | Yes |
| 8 | Term | AGA | Female | 3215 | 17 | 174 | 68 | 32 | Yes | Yes |
| 9 | Term | AGA | Male | 3840 | 35 | 160 | 108.6 | 0.9 | Yes | Yes |
| 13 | Term | AGA | Female | 2720 | 36 | 160 | 66 | 4 | Yes | Yes |
| 14 | Term | IUGR | Female | 3630 | 31 | 172 | 65 | 14.1 | Yes | Yes |
| 15 | Term | AGA | Female | 3380 | 36 | 157 | 68 | 12 | Yes | Yes |
| 16 | Term | AGA | Female | 1980 | 35 | 170 | 69 | 8 | Yes | Yes |
| 18 | Term | AGA | Female | 3760 | 28 | 150 | 45 | 12 | Yes | Yes |
| 20 | Term | AGA | Male | 3670 | 34 | 170 | 74 | 15 | Yes | Yes |
| 21 | Term | AGA | Female | 3280 | 25 | 157 | 58 | 10 | Yes | Yes |
| 22 | Term | AGA | Male | 3480 | 25 | NA | NA | NA | Yes | Yes |
| 23 | Term | AGA | Male | 1535 | 34 | 167 | 68 | 15 | Yes | Yes |
| 26 | PTM | AGA | Male | 184 | 35 | 155 | 55 | 17 | Yes | Yes |
| 34 | Term | AGA | Male | 1830 | 24 | 159 | 85 | 7 | Yes | Yes |
| 36 | Term | AGA | Male | 940 | 30 | 167 | 54 | 10 | Yes | Yes |
| 37 | Term | IUGR | Female | 3590 | 34 | 156 | 73 | 11 | Yes | Yes |
| 41 | PTE | AGA | Male | 1700 | 31 | 162 | 112 | 4 | Yes | Yes |
| 42 | PTE | AGA | Male | 1620 | 34 | 176 | 62 | 11 | Yes | Yes |
| 43 | PTE | AGA | Male | 860 | 34 | 176 | 62 | 11 | Yes | Yes |
| 44 | Term | IUGR | Male | 990 | 31 | 176 | 110 | 8 | Yes | Yes |
| 45 | Term | SGA | Female | 2410 | 22 | 169 | 60 | 21 | Yes | Yes |
| 47 | PTE | IUGR | Male | 2790 | 37 | 165 | 56 | 8 | Yes | Yes |
| 49 | PTE | AGA | Male | 1690 | 24 | 170 | 50 | 6 | Yes | Yes |
| 50 | PTM | AGA | Female | 3390 | 32 | 163 | 70 | 14 | Yes | Yes |
| 51 | Term | IUGR | Female | 3250 | 40 | 151 | 40 | 7 | Yes | Yes |
| 52 | PTL | IUGR | Female | 2240 | 39 | NA | NA | NA | Yes | Yes |
| 53 | PTE | PE | Male | 1120 | 27 | NA | NA | NA | Yes | Yes |
| 54 | Term | SGA | Male | 2000 | 33 | 159 | 54 | 13 | Yes | Yes |
| 55 | Term | IUGR | Male | 2350 | 30 | 161 | 63 | -1 | Yes | Yes |
| 56 | Term | AGA | Male | 2800 | 32 | 164 | 57 | 11 | Yes | Yes |
| 58 | PTE | AGA | Male | 2150 | 46 | 163 | 61 | 8 | Yes | Yes |
| 59 | PTE | IUGR | Male | 3840 | 46 | 163 | 61 | 8 | Yes | Yes |
| 62 | PTE | AGA | Female | 1870 | 37 | 165 | 82 | 9 | Yes | Yes |
| 66 | Term | SGA | Male | NA | 39 | NA | NA | NA | Yes | Yes |
| 67 | PTM | AGA | Male | 3325 | 39 | NA | NA | NA | No | Yes |
| 68 | PTE | IUGR | Female | 700 | 26 | NA | NA | NA | No | Yes |
| 69 | NA | AGA | Female | 565 | 40 | 170 | 62 | 8 | Yes | Yes |
| 71 | PTL | PE | Female | 2105 | 25 | 157 | 53 | 13 | Yes | Yes |
| 72 | PTM | AGA | Female | 3300 | 28 | 171 | 62 | 16 | Yes | Yes |
| 73 | PTL | IUGR | Female | 1425 | 24 | 171 | 69 | 11 | Yes | Yes |
| 74 | PTE | AGA | Male | 1290 | 34 | 158 | 54 | 9 | Yes | Yes |
| 75 | PTE | AGA | Male | 2380 | 24 | 156 | 73 | 9 | Yes | Yes |
| 78 | PTL | IUGR | Female | 2800 | 34 | 171 | 75 | 15 | Yes | Yes |
| 79 | Term | PE | Female | 3670 | 25 | 164 | 55 | 21 | Yes | Yes |
| 80 | PTM | PE | Female | 1800 | 28 | 163 | 58 | 10 | Yes | Yes |
| 87 | PTE | PE | Female | 960 | 31 | 171 | 70 | 16 | Yes | Yes |
| 88 | Term | IUGR | Male | 2030 | 22 | 143 | 47 | 13 | Yes | Yes |
| 89 | PTM | AGA | Male | NA | 34 | 164 | 50 | 11 | Yes | Yes |
| 90 | PTM | IUGR | Male | NA | 34 | 164 | 50 | 11 | Yes | Yes |
| 91 | PTE | IUGR | Female | NA | 34 | 160 | 80 | 5 | Yes | Yes |
| 93 | PTM | AGA | Female | NA | 35 | 162 | 50 | NA | Yes | Yes |
| 94 | PTM | AGA | Female | NA | 36 | 165 | 57 | 12 | Yes | Yes |
| 97 | PTM | AGA | Male | NA | 43 | 159 | 60 | 14 | Yes | Yes |
| 98 | PTL | IUGR | Male | 1680 | 37 | 162 | 65 | 6 | Yes | Yes |
| 142 | PTL | IUGR | Female | 2030 | 41 | 166 | 49 | 20 | No | Yes |
| 143 | PTM | SGA | Female | 1700 | 32 | NA | NA | NA | Yes | Yes |
| 144 | PTM | PE | Female | 620 | 32 | NA | NA | NA | Yes | Yes |
| 146 | Term | AGA | Male | 2320 | 34 | NA | NA | NA | Yes | Yes |
| 147 | PTM | AGA | Female | 1530 | 34 | 160 | 67 | 8 | No | Yes |
| 152 | PTL | PE | Female | 1920 | 41 | 161 | 100 | 6 | Yes | Yes |
| 154 | PTL | IUGR | Male | 2510 | 31 | 158 | 44 | 12 | No | Yes |
| 155 | PTL | IUGR | Female | 1960 | 35 | NA | NA | NA | Yes | Yes |
| 158 | PTL | IUGR | Female | 1340 | 32 | 172 | 60 | 22.7 | Yes | Yes |
| 159 | PTL | AGA | Female | 1770 | 32 | 172 | 60 | 22.7 | Yes | Yes |
| 160 | Term | IUGR | Female | 1450 | 38 | 154 | 46 | 8.2 | Yes | Yes |
| 161 | Term | IUGR | Female | 1670 | 31 | 160 | 68 | 11.7 | Yes | Yes |
| 162 | PTM | AGA | Female | 2210 | 37 | 166 | 56 | 9 | Yes | Yes |
| 163 | PTL | AGA | Female | 2490 | 30 | 177 | 78 | 16.5 | Yes | Yes |
| 164 | PTL | AGA | Male | 2520 | 30 | 177 | 78 | 16.5 | Yes | Yes |
| 165 | PTL | AGA | Female | 2120 | 30 | 177 | 78 | 16.5 | Yes | Yes |
| 166 | PTE | AGA | NA | 1150 | NA | NA | NA | NA | Yes | Yes |
| 167 | PTM | PE | NA | 810 | NA | NA | NA | NA | Yes | Yes |
| 170 | PTL | AGA | Male | 1345 | 44 | NA | NA | NA | Yes | Yes |
| 186 | PTE | PE | Female | 1330 | 25 | 173 | NA | NA | Yes | Yes |
| 191 | Term | AGA | Male | 2740 | 41 | NA | NA | NA | No | Yes |
| 216 | Term | AGA | Male | 2370 | 33 | 160 | 62 | 17 | Yes | Yes |
| 217 | PTE | IUGR | NA | 2270 | NA | NA | NA | NA | Yes | Yes |
| 222 | PTE | AGA | NA | 1980 | NA | NA | NA | NA | Yes | Yes |
| 225 | PTE | AGA | NA | 2740 | NA | NA | NA | NA | Yes | Yes |
| 226 | Term | AGA | NA | 2690 | NA | NA | NA | NA | Yes | Yes |

**Supplemental Table 2**. Summary statistics for the clinical characteristics of the placenta samples used for qRT-PCR and pyrosequencing. Term (37 > weeks), PTL – preterm late (35 to 37 weeks), PTM – preterm moderate (32 to 35 weeks), PTE – preterm extreme (<32 weeks).

| **Pyrosequencing placental cohort summary:** | | | **qRT-PCR placental cohort summary:** | | |
| --- | --- | --- | --- | --- | --- |
| **Placenta** |  |  | **Placenta** |  |  |
| Gestational age | PTE | 18 | Gestational age | PTExt | 19 |
|  | PTM | 13 |  | PTMod | 15 |
|  | PTL | 13 |  | PTLeve | 15 |
|  | Term | 32 |  | Term | 33 |
|  | NA | 1 |  | NA | 1 |
| Child's phenotype | AGA | 45 | Child's phenotype | AGA | 48 |
|  | IUGR | 19 |  | IUGR | 22 |
|  | SGA | 4 |  | SGA | 4 |
|  | PE | 9 |  | PE | 9 |
| Sex | Male | 31 | Sex | Male | 35 |
|  | Female | 40 |  | Female | 42 |
|  | NA | 6 |  | NA | 6 |
| Birth weight (g) | Min | 184 | Birth weight (g) | Min | 184 |
|  | Mean | 2194 |  | Mean | 2182 |
|  | Median | 2135 |  | Median | 2135 |
|  | Max | 3840 |  | Max | 3840 |
|  | NA | 7 |  | NA | 7 |
| Mother |  |  | Mother |  |  |
| Mother's age | Min | 17 | Mother's age | Min | 17 |
|  | Mean | 32.21 |  | Mean | 32.36 |
|  | Median | 33 |  | Median | 33 |
|  | Max | 46 |  | Max | 46 |
|  | NA | 6 |  | NA | 6 |
| Mother's hight | Min | 143 | Mother's hight | Min | 143 |
|  | Mean | 164.1 |  | Mean | 164 |
|  | Median | 164 |  | Median | 163.5 |
|  | Max | 177 |  | Max | 177 |
|  | NA | 16 |  | NA | 19 |
| Mother's weight | Min | 40 | Mother's weight | Min | 40 |
|  | Mean | 64.33 |  | Mean | 64.9 |
|  | Median | 62 |  | Median | 62 |
|  | Max | 112 |  | Max | 112 |
|  | NA | 17 |  | NA | 20 |
| Mother's gained weight | Min | -1 | Mother's gained weight | Min | -1 |
|  | Mean | 11.83 |  | Mean | 11.92 |
|  | Median | 11 |  | Median | 11 |
|  | Max | 32.00 |  | Max | 32.00 |
|  | NA | 18 |  | NA | 21 |
