# Supplementary material for: PIK3R1 and G0S2 are human placenta-specific imprinted genes associated with germline-inherited maternal DNA methylation
Source: Epigenetics. 2025 Jun 26;20(1):2523191. doi: 10.1080/15592294.2025.2523191 (PMC12203861; doi:10.1080/15592294.2025.2523191)
Supplement: Supplemental Material [file KEPI_A_2523191_SM1272.zip › Supplementary files/Supplemental_Table_3.docx]

**Supplemental Table 3.** List of PCR primers used in this study.

| **Gene** | **Variants** | **Oligo name** | **Sequence (5’ -> 3’)** |
| --- | --- | --- | --- |
| **Genotyping (DNA)** | | | |
| *PIK3R1* | rs138814985, rs2888323 | Forward | gttggcttctcaatgaggag |
|  |  | Reverse | AATCCCCAAAGCTGTTCTTCCA |
|  |  | Sequencing Reverse | GCTGTTCTTCCACCAAGTG |
|  | rs3730089 | Forward | tccatattgcatggaattgtgaact |
|  |  | Reverse | CTCCCCAGTACCATTCAGCATC |
| *G0S2* | rs1815548, rs932375 | Forward | TGGGACCTTCGCGTGCACACT |
|  |  | Reverse | GCTCTCCCAGTTGGAGACTCCG |
| **Bisulphite PCR** | | | |
| *PIK3R1* | rs138814985, rs2888323 | Forward | AGTTGGTTTTTTAATGAGGA |
|  |  | Outer Reverse | CCCTTTAAAATACCTATATCC |
|  |  | Inner Reverse | CCACCAAATAAACCAAACCCC |
| *G0S2* | rs1815548, rs932375 | Forward | GTTGTAGTTTTTTTAGTTGGAG |
|  |  | Outer Reverse | TCCCTAAACTCCGAATCCTCCCCT |
|  |  | Inner Reverse | TACACACTAACCTTCCCAC |
|  |  | Sequencing Forward | GATTGTGTGAGTTAGGGGGT |
| **Pyrosequencing** | | | |
| *PIK3R1* | rs138814985, rs2888323 | Forward (Sequencing) | AGTTGGTTTTTTAATGAGGA |
|  |  | Outer Reverse Bio | [Btn]CCCTTTAAAATACCTATATCC |
| *G0S2* | N/A | Forward | GTTGTAGTTTTTTTAGTTGGAG |
|  |  | Outer Reverse Bio | [Btn]TCCCTAAACTCCGAATCCTCCCCT |
|  |  | Inner Sequencing Forward | GATTGTGTGAGTTAGGGGGT |
| **Allelic RT-PCR** | | | |
| *PIK3R1* **(isoform 3)** | rs138814985, rs2888323, rs3730089 | Forward | CAATGAGGAGCCGGCAGTGAGC |
|  |  | Reverse | AGATATCTCCCCAGTACCATTCA |
| *PIK3R1* **(isoform 3)** **nested** RT-PCR (Out/Out) | rs138814985, rs2888323, rs3730089 | Forward | CAATGAGGAGCCGGCAGTGAGC |
|  |  | Reverse 2 | CTCCCCAGTACCATTCAGCATC |
| *PIK3R1* **(isoform 3)** **nested** RT-PCR (In/Out) | rs3730089 | Forward 2 | GGGAAACCGTTGAAATGCATAACCTG |
|  |  | Reverse 2 | CTCCCCAGTACCATTCAGCATC |
| *PIK3R1* **(isoform 3)** | rs3730089 | Forward 3 | TTTTTCATTGTCGGATACAGGCATT |
|  |  | Reverse 2 | CTCCCCAGTACCATTCAGCATC |
| *PIK3R1* **(isoform 3)** MACS fractions | rs138814985, rs2888323, rs3730089 | Forward 4 | gttggcttctcaatgaggag |
|  |  | Reverse | AGATATCTCCCCAGTACCATTCA |
| *PIK3R1* **(isoform 3)** | rs3730089 | Forward 3 (Sequencing) | TTTTTCATTGTCGGATACAGGCATT |
| *PIK3R1* **(isoform 3)** | rs138814985, rs2888323 | Reverse 3 (Sequencing) | AATCCCCAAAGCTGTTCTTCCA |
| *PIK3R1* **(isoform 1)** | rs3730089 | Forward 5 | TCTCTGAAATTTTCAGCCCTATGCT |
|  |  | Reverse 2 | CTCCCCAGTACCATTCAGCATC |
| *PIK3R1* **(isoform 1)** | rs3730089 | Forward 5 (Sequencing) | TCTCTGAAATTTTCAGCCCTATGCT |
| *G0S2* **nested** RT-PCR (Out/ Out) | rs932375 | Forward | GCTCTGACCGCGCTGGCCTGG |
|  |  | Outer Reverse | GAGGCGGGAATGACCTTAGTGG |
| *G0S2* **nested** RT-PCR (Out/ In) |  | Forward | GCTCTGACCGCGCTGGCCTGG |
|  |  | Inner Reverse | GAATGACCTTAGTGGCACGGCGCGAG |
| **qRT-PCR SYBR™ Green** | | | |
| *ACTB* | N/A | Forward | CCGGCTTCGCGGGCGACGAT |
|  |  | Reverse | CTCCATGTCGTCCCAGTTGG |
| *RPL19* | N/A | Forward | AATCGCCAATGCCAACTCCCGTCA |
|  |  | Reverse | CCTATGCCCATGTGCCTGCCCTTC |
| *PIK3R1* isoform 3 | N/A | Forward | GGGAAACCGTTGAAATGCATAACCTG |
|  |  | Reverse | GTTTTTCATTCACTTCTTCCCTCGAG |
| *PIK3R1* isoform 1 | N/A | Forward | TCTCTGAAATTTTCAGCCCTATGCT |
|  |  | Reverse | GTCGTTCATTCCATTCAGTTGAG |
| *PIK3R1* all isoforms | N/A | Forward | AGCTATTGAAGCATTTAATGAAACCA |
|  |  | Reverse | CACTGATTCGAGACTTCAACTTATC |
| *KRT7* | N/A | Forward | CAGGCTGAGATCGACAACATC |
|  |  | Reverse | CTTGGCACGAGCATCCTT |
| *VIM* | N/A | Forward | GGCTCAGATTCAGGAACAGC |
|  |  | Reverse | AGCCTCAGAGAGGTCAGCAA |
| *CGB3* | N/A | Forward | GTGTCGAGCTCACCCCAGCATCCTA |
|  |  | Reverse | AGCAGCCCCTGGAACATCT |
| *COL3A1* | N/A | Forward | GGAGCTGGCTACTTCTCGC |
|  |  | Reverse | GGGAACATCCTCCTTCAACAG |
| *CD45* | N/A | CD45/PTPRC F | AGCTAAGGCGACAGAGATGCCTGA |
|  |  | CD45/PTPRC R | CTCACTGGGTGGATCCCTTTTCTTC |
| *CD14* | N/A | Forward | CGGAAGACTTATCGACCATGGAGC |
|  |  | Reverse | AAGGCTTCGGACCAGTCGGGCTGA |
| **TaqMan™** | | | |
| *G0S2* | N/A | Assay Id: Hs00377852_g1 (G0S2) TaqMan Gene Expression Assay (FAM) | |
| *RPL19* | N/A | Assay Id Hs02338565_gH (RPL19) TaqMan Gene Expression Assay | |
| **Cloning** | | | |
| pGEM®-T Easy Vector | N/A | Forward | GATGGTGCTGCAAGGCGATTAAGTTG |
|  |  | Reverse | ATGTTGTGTGGAATTGTGAGCGGA |
|  |  | Sp6 primer | ATTTAGGTGACACTATAG |
|  |  | MF13 Forward | GTAAAACGACGGCCAG |
|  |  | Seq(S) T7 | TAATACGACTCACTATAGGG |

Mouse genes

| **Gene** | **Variants** | **Name** | **Sequence (5’ -> 3’)** |
| --- | --- | --- | --- |
| **Bisulphite PCR** | | | |
| *Pik3r1* | N/A | Outer Forward | TATTAAGTGGTTTTAGTTTTTGAG |
|  |  | Outer Reverse | TTAACTTAAATACCCCTCCCCCT |
|  |  | Inner Forward | GTAAAGAATTTAGTTGGAGGAGAG |
|  |  | Inner Reverse | TAACTCAACAAATATTTAAACCT |
| *G0s2* | rs31626975 | Outer Forward | AGTTAAGAAAGTAGTATTTTGGAAGA |
|  |  | Outer Reverse | CCAAAAAAATAACCACrAATAATAC |
|  |  | Inner Forward | TTTTGATTGGTGAGAGGTGATTTTT |
|  |  | Inner Reverse | CTAAAAACCCAAAACACCACTTC |
| **RT-PCR** | | | |
| *Pik3r1* | rs37236366, rs13463306 | Forward | ATTATGCATAACCATGATAAGCTGA |
|  |  | Reverse | CGGTTGCTGCTCCCGACATTCCAC |
