# Supplementary material for: PIK3R1 and G0S2 are human placenta-specific imprinted genes associated with germline-inherited maternal DNA methylation
Source: Epigenetics. 2025 Jun 26;20(1):2523191. doi: 10.1080/15592294.2025.2523191 (PMC12203861; doi:10.1080/15592294.2025.2523191)
Supplement: Supplemental Material [file KEPI_A_2523191_SM1272.zip › Supplementary files/Supplemental_Table_4.docx]

**Supplementary Table 4**

The number of heterozygous placenta samples used to determine allelic methylation and expression for *G0S2* and *PIK3R1*.

| **Variant** | **Sample** | **Genotype** | **Mother’s genotype** | **Father’s genotype** | **Methylation-sensitive genotyping (HpaII)** | | **Allelic expression** | |  |
| --- | --- | --- | --- | --- | --- | --- | --- | --- | --- |
| rs1815548 | BCN 5 | C/T | C/T | - | Pref. C | Pref. monoallelic | - | - |  |
|  | BCN 7 | C/T | C/T | - | T | Monoallelic | - | - |  |
|  | BCN 44 | C/T | C/T | - | T | Monoallelic | - | - |  |
|  | BCN 70 | C/T | C/T | - | Pref. C | Pref. monoallelic | - | - |  |
|  | BCN 95 | C/T | T | - | T | Maternal | - | - |  |
|  | 22BR 162 | C/T | - | - | T | Monoallelic | - | - |  |
|  | 21BR 311 | C/T | C/T | - | C | Monoallelic | - | - |  |
| rs932375 | BCN 12 | C/G | C | - | C | Maternal | C | Maternal |  |
|  | BCN 31 | C/G | C | - | C | Maternal | G | Paternal |  |
|  | 23BR 128 | C/G | C/G | C | G | Maternal | C | Paternal |  |
|  | 22BR 162 | C/G | - | - | C | Monoallelic | Pref. C | Pref. monoallelic |  |
|  | 23BR 294 | C/G | C | - | Pref. C | Pref. maternal | C | Maternal |  |
|  | 21BR 311 | C/G | C | - | Pref. C | Pref. maternal | C | Maternal |  |
|  | 21BR 430 | C/G | C/G | - | G | Monoallelic | C | Monoallelic |  |
|  | 21BR 432 | C/G | C | - | C | Maternal | Pref. C | Pref. Maternal |  |

**Summary for *PIK3R1* isoform 3 placenta-specific mDMR**

(n - a number of copies of the repeat).

| **Variant** | **Sample** | **Genotype** | **Mother’s genotype** | **Methylation-sensitive genotyping (HpaII)** | | **Allelic expression** | |  |
| --- | --- | --- | --- | --- | --- | --- | --- | --- |
| rs138814985 | BCN 5 | 3n/4n | 3n/4n | 3n | Monoallelic | - | - | |
|  | BCN 8 | 3n/4n | 3n | 3n | Maternal | 3n/4n | Biallelic | |
|  | BCN 60 | 3n/4n | - | 3n | Monoallelic | 4n | Monoallelic | |
|  | BCN 65 | 3n/4n | 3n/4n | 3n | Monoallelic | N/A | - | |
|  | BCN 70 | 3n/4n | 3n/4n | 3n | Monoallelic | 3n/4n | Biallelic | |
|  | BCN 92 | 3n/4n | 3n | 3n | Maternal | 3n/4n | Biallelic | |
|  | BCN 95 | 3n/4n | 3n | 3n | Maternal | - | - | |
|  | 22BR 160 | 3n/4n | 3n/4n | 4n | Monoallelic | - | - | |
|  | 22BR 161 | 3n/4n | 3n/4n | 4n | Monoallelic | 3n/4n | Biallelic | |
|  | 22BR 162 | 3n/4n | 3n | 3n | Maternal | 4n | Paternal | |
|  | 22BR 546 | 3n/4n | - | - | - | 3n/4n | Biallelic | |
| rs2888323 | BCN 6 | A/G | A | A | Maternal | G/A | Biallelic | |
|  | BCN 45 | A/G | A/G | A | Monoallelic | - | - | |
|  | BCN 46 | A/G | A | A | Maternal | - | - | |
|  | BCN 77 | A/G | - | Pref. A | Pref. monoallelic | G | Monoallelic | |
|  | 22BR 161 | A/G | A/G | A | Monoallelic | A/G | Biallelic | |
|  | 22BR 293 | A/G | A/G | A | Monoallelic | A/G | Biallelic | |
|  | 22BR 546 | A/G | - | - | - | A/G | Biallelic | |
|  | 22BR 548 | A/G | - | A/G | Biallelic | A/G | Biallelic | |
| rs3730089 | BCN 6 | G/A | G/A | - | - | G/A | Biallelic | |
|  | BCN 8 | G/A | G | - | - | A/G | Biallelic | |
|  | BCN 21 | G/A | G/A | - | - | Pref. G | Pref. monoallelic | |
|  | BCN 26 | G/A | G/A | - | - | Pref. G | Pref. monoallelic | |
|  | BCN 44 | G/A | G/A | - | - | G/A | Biallelic | |
|  | BCN 64 | G/A | G | - | - | G/A | Biallelic | |
|  | BCN 95 | G/A | G/A | - | - | G/A | Biallelic | |
|  | 22BR 161 | G/A | G/A | - | - | Pref. G | Pref. monoallelic | |
|  | 22BR 162 | G/A | G | - | - | A | Paternal | |
|  | 22BR 701 | G/A | G/A | - | - | G | Monoallelic | |

**Summary for the number of heterozygous fetal sample sets used to determine allelic methylation and expression for *G0S2* and *PIK3R1*.** CS - Carnegie stage

| Gene | Sample | Gestational age | Methylation | | Allelic expression | |  |
| --- | --- | --- | --- | --- | --- | --- | --- |
| *GOS2* | 14468 muscle | CS10 |  | Unmethylated |  | - |  |
|  | 14468  placenta | CS10 |  | DMR |  | - |  |
|  | 18425  muscle | 18-weeks |  | - |  | Biallelic |  |
|  | 18425  placenta | 18-weeks |  | - |  | Monoallelic |  |
| *PIK3R1* | 18425  placenta | 18-weeks |  | DMR |  | - |  |
|  | 14468  placenta | CS10 |  | DMR |  | - |  |
|  | 14468  brain | CS10 |  | Unmethylated |  | - |  |
|  | 18445  placenta | 18-weeks |  | - |  | Monoallelic |  |
|  | 18445  brain | 18- weeks |  | - |  | Biallelic |  |
