# Supplementary figures and images for: PIK3R1 and G0S2 are human placenta-specific imprinted genes associated with germline-inherited maternal DNA methylation
Source: Epigenetics. 2025 Jun 26;20(1):2523191. doi: 10.1080/15592294.2025.2523191 (PMC12203861; doi:10.1080/15592294.2025.2523191)

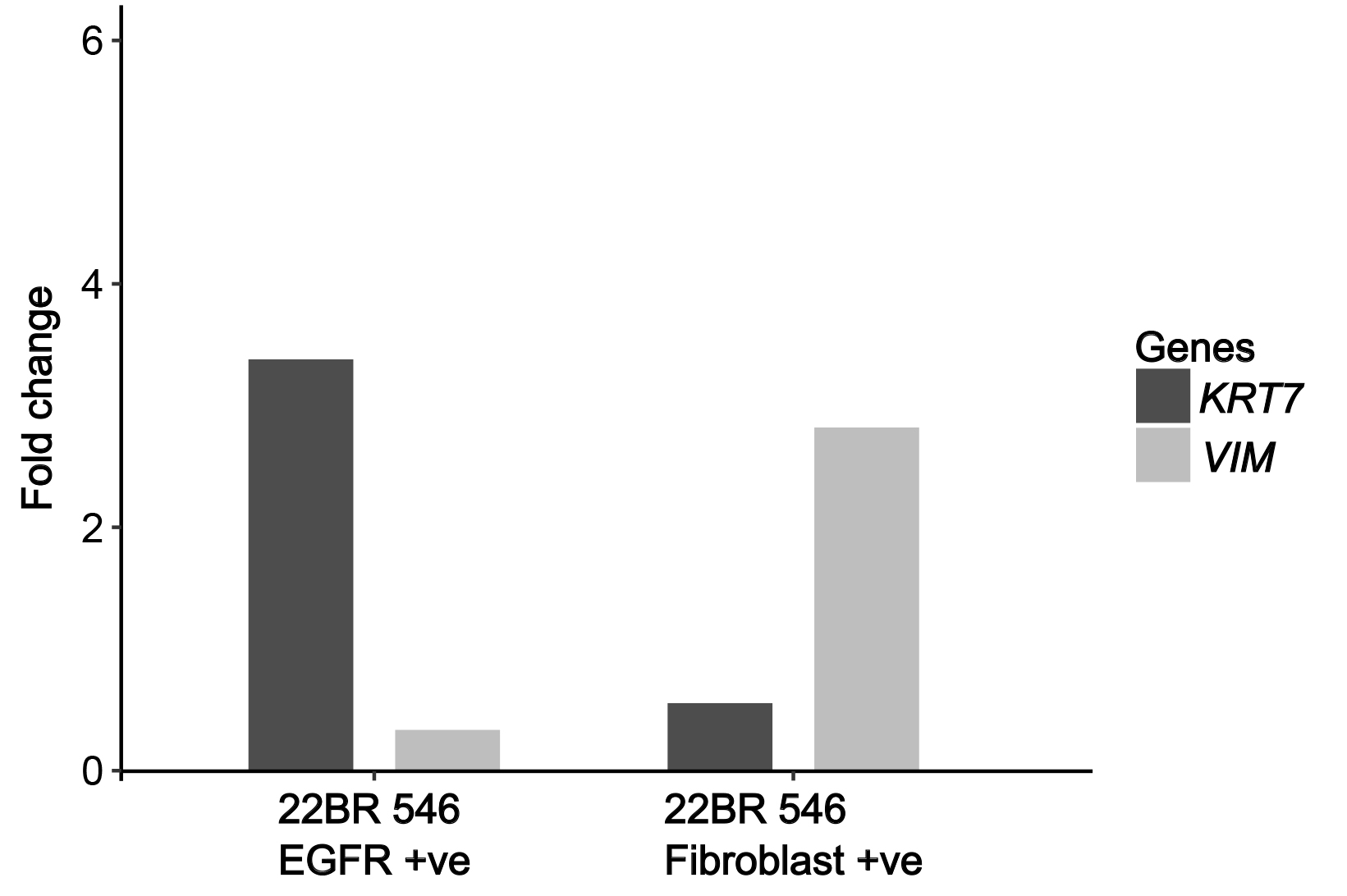

Supplement: Supplemental Material [file KEPI_A_2523191_SM1272.zip › Supplementary files/Supplemental_Figure_1_copy.jpg]

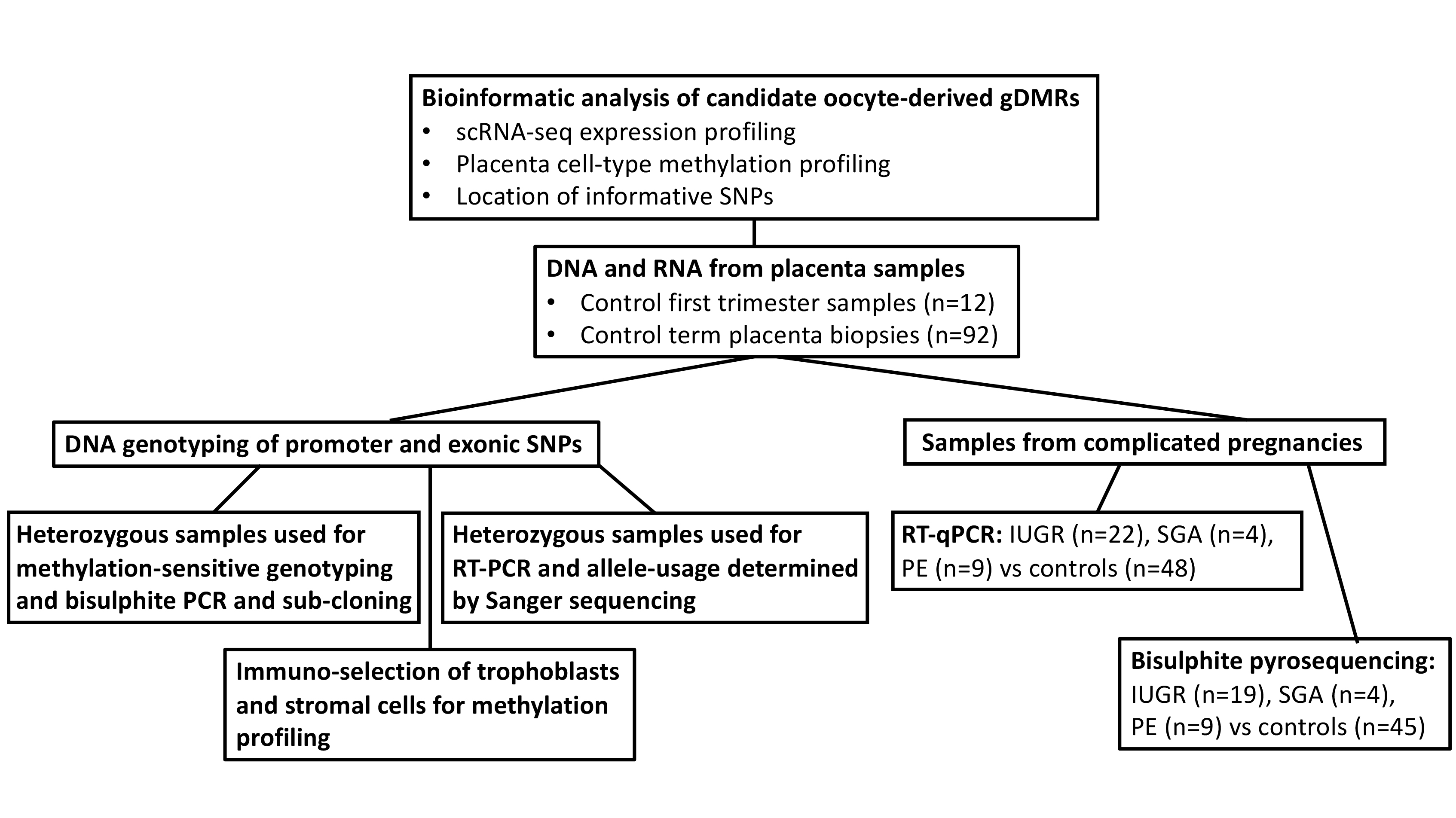

Supplement: Supplemental Material [file KEPI_A_2523191_SM1272.zip › Supplementary files/Supplemental_Figure_2_copy.jpg]

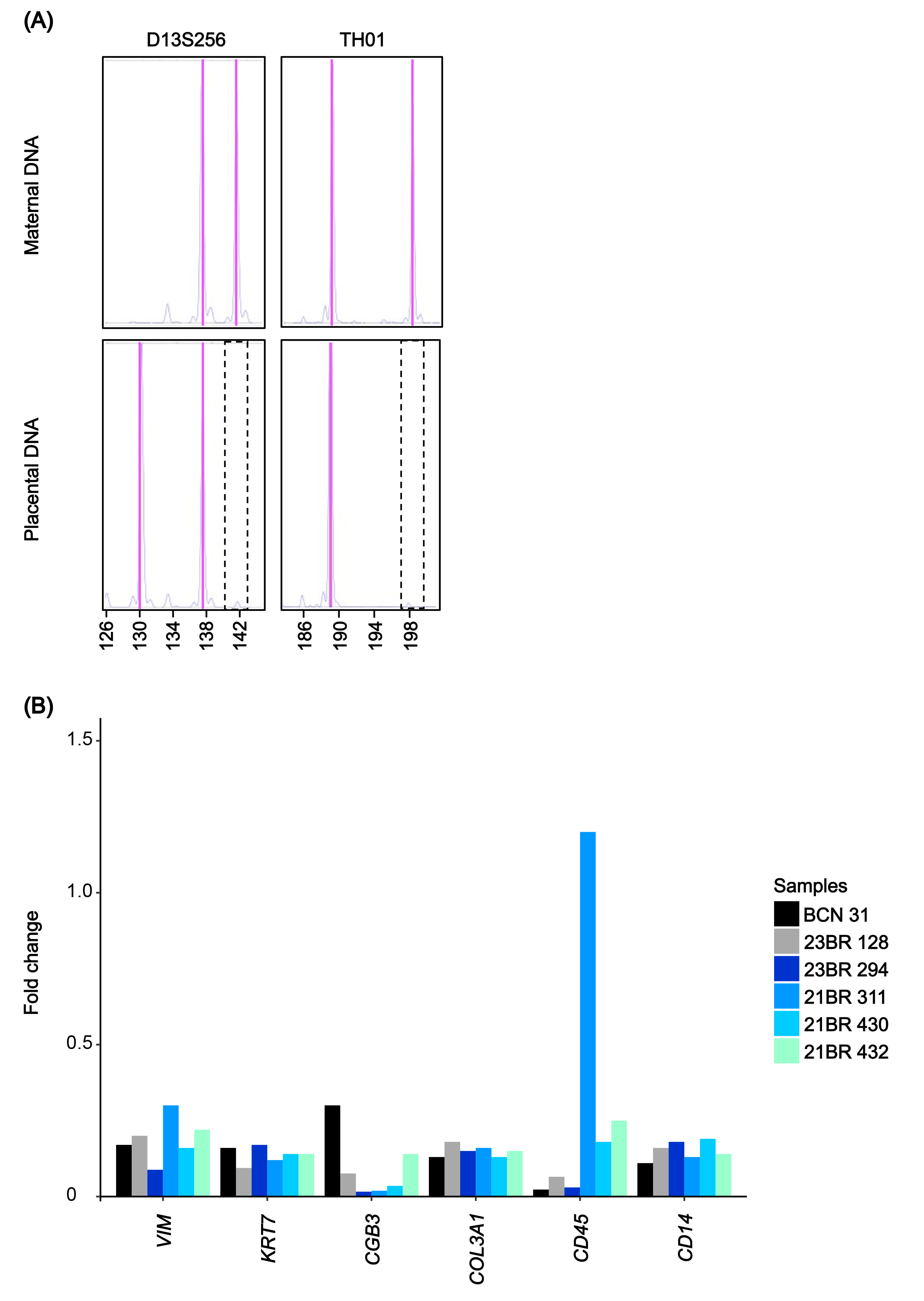

Supplement: Supplemental Material [file KEPI_A_2523191_SM1272.zip › Supplementary files/Supplemental_Figure_3_copy.jpg]

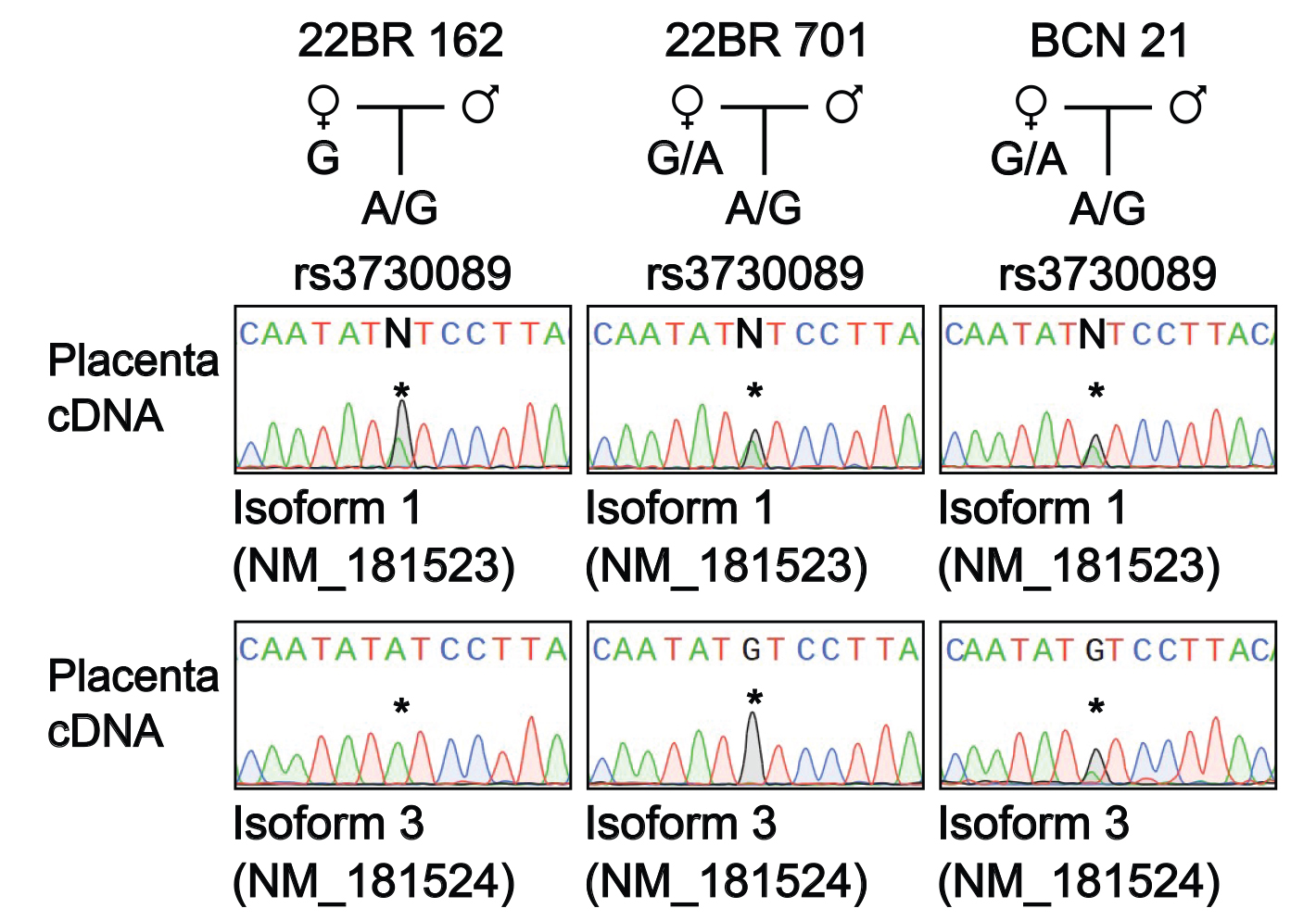

Supplement: Supplemental Material [file KEPI_A_2523191_SM1272.zip › Supplementary files/Supplemental_Figure_4_copy.jpg]

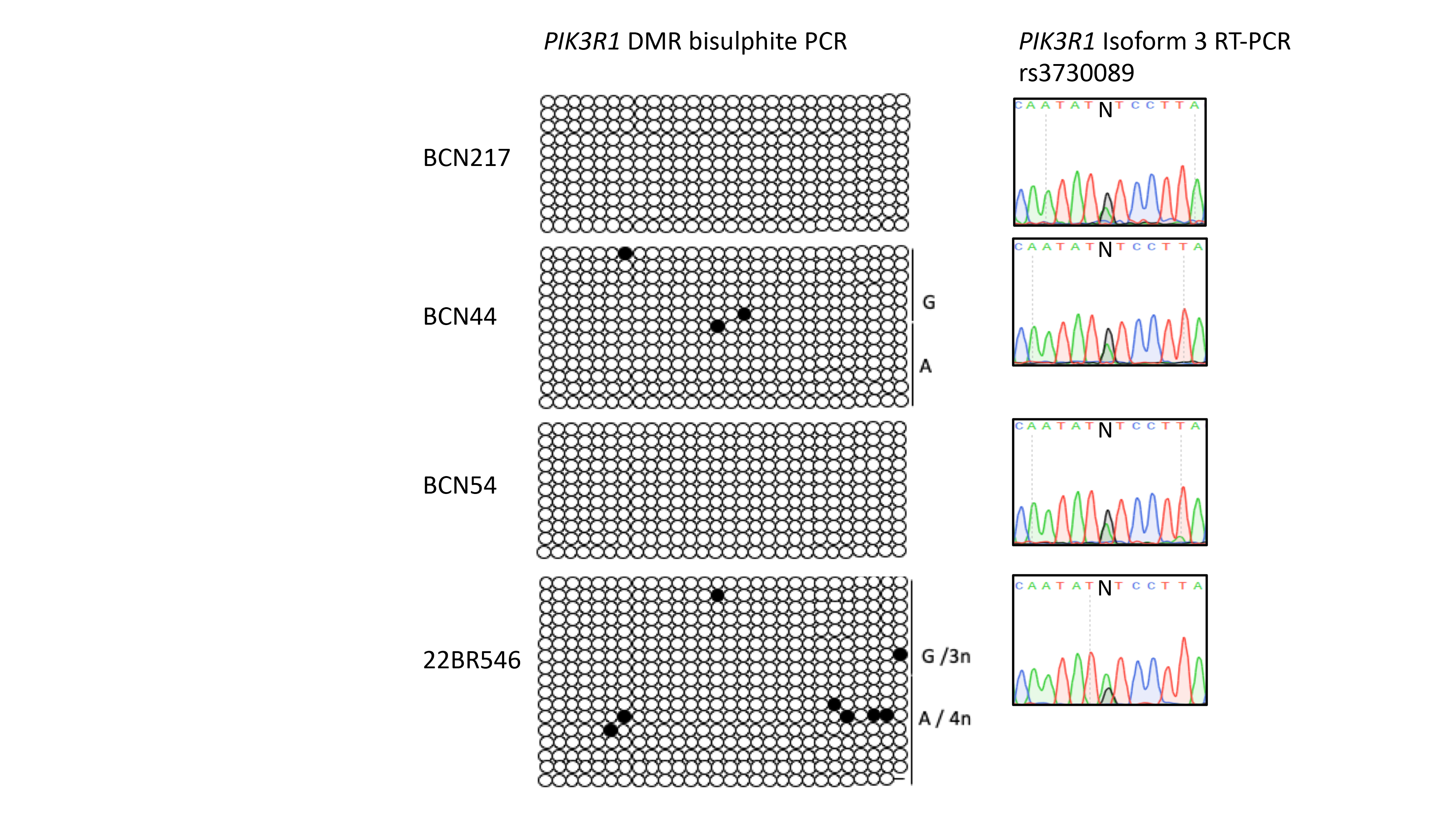

Supplement: Supplemental Material [file KEPI_A_2523191_SM1272.zip › Supplementary files/Supplemental_Figure_5_copy.jpg]
